# Safranal Ameliorates Renal Damage, Inflammation, and Podocyte Injury in Membranous Nephropathy via SIRT/NF-κB Signalling

**DOI:** 10.1007/s11596-025-00020-8

**Published:** 2025-03-04

**Authors:** Yan Bao, Ya-mei Ge, Zheng Wang, Hong-yun Wang, Qiong Wang, Jun Yuan

**Affiliations:** 1 Department of Nephrology, Renmin Hospital of Wuhan University, Wuhan, 430060 China; 2 The First Clinical College, Hubei University of Chinese Medicine, Wuhan, 430065 China; 3 BSc Biochemistry, University College London, London, WCIE 6BT UK

**Keywords:** Inflammation, Membranous nephropathy, Nuclear factor kappa B, Safranal, Sirtuin type-1

## Abstract

**Objective:**

Safranal is a natural product from saffron (*Crocus sativus* L.) with anti-inflammatory and nephroprotective potential. This study aimed to explore the role of safranal in a cationic bovine serum albumin (C-BSA)-induced rat model of membranous glomerulonephritis (MGN).

**Methods:**

After model establishment, Sprague–Dawley rats were administered 100 or 200 mg/kg safranal by gavage. A biochemical analyser was used to measure the urine protein levels and serum levels of renal function parameters. Hematoxylin–eosin and immunofluorescence staining of kidney tissues were performed to examine histopathological changes and assess the expression of IgG, C3, and Sirt1. Western blotting was performed to measure the protein levels of podocin, nephrin, Sirt1, and factors involved in the NF-κB/p65 pathway. Inflammatory cytokine levels in renal homogenates were determined by ELISA.

**Results:**

Safranal at 100 or 200 mg/kg reduced kidney weight (2.07 ± 0.15 g and 2.05 ± 0.15 g) and the kidney somatic index (0.83 ± 0.08% and 0.81 ± 0.08%) in MGN rats compared with those in the model group without drug administration (2.62 ± 0.17 g and 1.05 ± 0.1%). C-BSA increased the urine protein level to 117.68 ± 10.52 mg/day (compared with the sham group, 5.03 ± 0.45 mg/day), caused dysregulation of renal function indicators, and induced glomerular expansion and inflammatory cell infiltration in the rat kidney samples. All the biochemical and histological changes were improved by safranal administration. Safranal at two doses also increased the fluorescence intensities of IgG (0.1 ± 0.009 and 0.088 ± 0.008) and C3 (0.065 ± 0.006 and 0.048 ± 0.004) compared with those in the MGN group (0.15 ± 0.013 and 0.086 ± 0.008). Additionally, safranal reversed the downregulation of podocin, nephrin, and Wilms tumor protein-1 (WT1) levels and reversed the high inflammatory cytokine levels in MGN rats. Mechanistically, safranal activated Sirt1 signalling to interfere with NF-κB signalling in the kidney tissues of MGN rats.

**Conclusions:**

Safranal ameliorates renal damage, inflammation, and podocyte injury in MGN by upregulating SIRT1 and inhibiting NF-κB signalling.

## Introduction

Membrane nephropathy is a glomerulopathy that primarily affects podocytes and compromises the integrity of the glomerular basement membrane [1]. It is also known as membranous glomerulonephritis (MGN), becoming a leading cause of pathological nephropathy in adults [2]. Approximately 40% of patients ultimately progress to end-stage renal failure within 10–15 years [3]. The characteristics of MGN include a progressive decline in renal function, podocyte injury, sustained inflammation, oxidative stress, and the development of glomerular and tubulointerstitial fibrosis [4]. Increasing evidence reveals that animal models of MGN exhibit glomerular injury, reactive oxygen species overproduction-induced oxidative stress, the stimulation of proinflammatory factors, and the diminishment of antioxidant compounds [2, 5]. In this study, Sprague–Dawley rats injected with cationic bovine serum albumin (C-BSA) for 4 consecutive weeks were used as the rat MGN model as described in previous studies [2, 5, 6].

An increasing number of studies indicate the advantages and promising prospects of natural compounds or traditional Chinese herbal medicines for MGN treatment [7–9]. For example, a traditional Chinese prescription, Yi Shen An, was reported to attenuate C-BSA-induced MGN in rats by inhibiting the formation of circulating immune complexes and removing the accumulation of IgG and complement C3 from the glomerular basement membrane [8]. Compound extracts from hydrangea paniculata improve renal function, inhibit IgG deposition and reduce glomerular lesions in a rat MGN model [10]. Safranal is a natural product from saffron (*Crocus sativus* L.) with biological activities, such as anti-inflammatory and antioxidative stress effects, on renal tissue in the context of type 2 diabetes and diabetic nephropathy [11, 12]. Safranal has also been reported to mitigate distal renal injury induced by ischemia/reperfusion [13] and to protect rats against cisplatin-induced oxidative injury and nephrotoxicity [14]. However, the functions of safranal in MGN are not fully understood.

Sirtuin type-1 (Sirt1)/nuclear factor kappa B (NF-κB) signalling is intricately associated with renal and podocyte injury in kidney diseases such as diabetic nephropathy [15, 16]. Sirt1 is a member of the silent information regulator 2-like family of proteins [17]. The silencing of Sirt1 in podocytes can induce severe podocyte injury and increase proteinuria in diabetic mice, and the overexpression of Sirt1 has the opposite effect [18]. Sirt1 can deacetylate NF-κB/p65 to inhibit the inflammatory response. The promotion of Sirt1 nuclear translocation and the inactivation of NF-κB are beneficial for the alleviation of diabetic nephropathy [15]. However, the role of Sirt1/NF-κB in MGN has not yet been reported. Importantly, a recent study reported that safranal interferes with NF-κB signalling by activating Sirt1 to increase p65 deacetylation and inactivating IKK to decrease IκBα degradation in osteoporosis [19]. Therefore, whether safranal can slow MGN progression by regulating Sirt1/NF-κB signalling was explored in this study.

This study aimed to investigate the effects of safranal on renal function, glomerular structural changes, IgG and C3 deposition, and podocyte protein levels and the underlying mechanism. This study may provide a novel compound with therapeutic potential for MGN.

## Materials and Methods

### Animals and Grouping

A total of 32 Sprague–Dawley rats (210 ± 10 g, 6‒8 weeks) were purchased from Beijing Vital River Animal Technology (China). The animals were maintained in a standard environment with a controlled temperature (20–24 °C), a 12 h/12 h light/dark cycle, and 45%–55% humidity. The protocol for the animal experiments in this study was approved by the Animal Ethics Committee of Wuhan Myhalic Biotechnology Co., Ltd. (No. HLK-202309078). All procedures were performed in line with the National Institutes of Health Guide for Laboratory Animal Care and Use. The rats were randomly divided into 4 experimental groups (*n* = 8/group): the sham group, MGN group, MGN + Safranal (Saf)-100 group, and MGN + Saf-200 group.

### Establishment of a Rat MGN Model and Animal Grouping

All the rats were allowed to adapt to the environment for 1 week. To establish the rat MGN model, 24 rats were administered C-BSA as previously described [2, 6, 20]. Briefly, for the preimmunization phase, 1 mg of C-BSA was dissolved in 0.5 mL of normal saline and emulsified with an equal amount of incomplete Freund’s adjuvant, resulting in the formation of a milky white suspension. The rats received multiple subcutaneous injections of C-BSA in the neck, groin, and armpit regions, with a volume of 0.1 mL per rat, which was administered once every other day for a total of 3 injections. During the immunization phase, C-BSA was combined with an equal volume of phosphate buffer followed by injection into the rat tail vein at a dosage of 16 mg/kg once every other day 3 times a week for 4 weeks. The MGN condition of the rats was confirmed by assessing 24-h proteinuria levels via a Bradford assay kit (Sigma-Aldrich, USA). Safranal (HY-N7560, MedChem Express, USA) was dissolved in distilled water and was administered by gavage to MGN rats for 4 consecutive weeks. The rats in the MGN + Saf-100 group or the MGN + Saf-200 group received 100 or 200 mg/kg safranal daily. The doses and administration of safranal were identified according to previous animal experiments focused on safranal [14, 21]. The rats in the sham group and MGN group were given distilled water via gavage.

### Collection of Rat Urine and Serum Samples and Biochemical Analysis

After 4 weeks, urine and serum samples were collected. The rats were placed in individual metabolic cages to collect urine within 24 h. All the rats fasted but had free access to water during this period. To remove the debris and collect the supernatants, the urine samples were centrifuged at 3000 rpm for 10 min. The supernatants were stored at − 80 °C until use. After anaesthesia, blood samples were collected from the abdominal aorta, followed by centrifugation at 3000 rpm for 10 min at 4 °C. The serum samples were stored at − 20 °C. An automatic biochemical analyser (Hitachi 7600, Japan) was used to measure the protein levels and parameters of the serum samples, which included the levels of albumin, blood urea nitrogen, serum creatine, total cholesterol, and triglycerides.

### Kidney Tissue Sampling

After the collection of urine and serum samples, the rats were euthanized by excessive anaesthesia by injection with 150 mg/kg pentobarbital sodium and cervical dislocation. Next, the kidney tissues of the rats were harvested. For histological analysis, a portion of the kidney tissue was fixed with 4% paraformaldehyde, and the remaining renal samples were frozen in liquid nitrogen and stored at − 80 °C.

### Hematoxylin and Eosin (H&E) Staining

The kidney samples fixed with 4% paraformaldehyde for 48 h were then dehydrated in ethanol, embedded in paraffin and cut into 5-μm thick sections. The sections were dewaxed with a series of ethanol (95%, 90%, 80% and 70%) and stained with 0.1% hematoxylin (Solarbio, China) for 10 min and 0.5% eosin (Solarbio, China) for 1 min. Morphological alterations were observed under a light microscope (Olympus, Japan).

### Periodic Acid-Schiff Staining

The kidney tissues were fixed with 4% paraformaldehyde overnight, dehydrated, and embedded in paraffin. Next, the samples were longitudinally sectioned at a thickness of 4 μm, followed by staining with periodic acid-Schiff (Sigma Aldrich, USA). An optical microscope (Olympus, Japan) was used to observe changes in kidney tissues. The percentage of the PAS-positive area was semiquantitatively analysed via Image-Pro Plus (Media Cybernetics, USA).

### Immunofluorescence Staining

Frozen sections of the renal cortex (5 μm) were fixed with 4% paraformaldehyde at 4 °C for 10 min and washed 3 times with phosphate-buffered saline. Next, the sections were blocked with 5% normal goat serum in 0.5% Triton X-100 for 30 min at ambient temperature. After that, the sections were incubated with goat anti-rat-IgG FITC antibody (ab6840; Abcam, UK) or anti-complement C3 FITC antibody (ab182890; Abcam, UK) at a dilution of 1:1000 for 1 h at 37 °C. The sections were then incubated with goat anti-mouse IgG Alexa Fluor 488 (#4408, Cell Signalling Technology, USA) or goat anti-rabbit IgG Alexa Fluor 555 (#4413, Cell Signalling Technology, USA) for 30 min at 37 °C. For immunofluorescence staining of Sirt1, the sections were incubated with anti-Sirt1 antibody (ab189494, Abcam, UK; dilution: 1:100) at 4 °C overnight, followed by incubation with goat anti-rabbit IgG H&L (ab150077, Abcam, UK; dilution: 1:1000) for 30 min at 37 °C. DAPI was then used to stain the sections at 5 μg/mL for 5 min. A confocal microscope (Leica, Germany) was used to observe the sections, and Image-Pro software (Media Cybernetics, USA) was used to determine the intensity of IgG, C3, or Sirt1.

### Immunohistochemical Staining of Wilms Tumor Protein-1 (WT-1)

To quantify changes in the number of podocytes in the kidneys of the rats, a rabbit anti-WT-1 polyclonal antibody (MA5-32215, Thermo Fisher Scientific, USA) was used to identify podocytes according to the manufacturer’s protocol. The kidney tissues were placed in 10% neutral buffered formalin for 24 h, embedded in paraffin, and cut into 4-μm-thick sections. Immunohistochemistry was performed on a Ventana Discovery immunostainer with a rabbit anti-WT-1 polyclonal antibody (Thermo Fisher Scientific, USA). The staining was visualized via a Warp Red Chromogen Kit (Biocare Medical, USA).

### Western Blotting

Proteins in renal cortex samples were extracted via radioimmunoprecipitation lysis buffer (Millipore, USA) and quantified via a bicinchoninic acid assay kit (Sigma Aldrich, USA). The proteins were separated via 10% SDS‒PAGE and transferred to 0.45 μm polyvinylidene difluoride membranes (Millipore, USA). Next, 5% bovine serum albumin was used to block the membranes for 1 h at ambient temperature. The membranes were incubated with primary antibodies (purchased from Abcam, UK or Cell Signalling Technology, USA) at 4 °C overnight. The primary antibodies used were against podocin (also known as NPHS2, ab181143, 1:2000), nephrin (ab216341, 1:1000), Sirt1 (ab110304, 1:1000), p-p65 (ab76302, 1:1000), p65 (#8242, 1:1000), p-IκB (#9242, 1:1000), IκB (#2859, 1:1000), and β-actin (#4967, 1:1000). After incubation with primary antibodies, the membranes were washed 3 times with TBST buffer (Procell, China), followed by incubation with secondary antibodies (Abcam) at ambient temperature for 1 h. An enhanced chemiluminescence kit (Millipore, USA) was used to visualize the bands with an imaging system. The signal intensity was analysed via ImageJ software (National Institutes of Health, USA).

### Enzyme-Linked Immunosorbent Assay (ELISA)

The concentrations of inflammatory mediators, including IL-6, IL-2, and TNF-α, in renal homogenates were measured via a rat IL-6 ELISA kit (ab234570, Abcam, UK), a rat IL-2 ELISA kit (ab221834, Abcam, UK), and a rat TNF-α ELISA kit (ab46070, Abcam, UK), respectively, following the manufacturers’ recommendations. The detection ranges of the corresponding kits for IL-6, IL-2, and TNF-α were 125–8000 pg/mL, 31.25–2000 pg/mL, and 31.25–1000 pg/mL, respectively.

### Statistical Analysis

The data generated in the study were analysed via GraphPad Prism (version 8.8). Differences among multiple groups were compared via one-way analysis of variance followed by Tukey’s post hoc test. The data are shown as the means ± standard deviations (SDs). A value of *P* < 0.05 was defined as the threshold for all the statistical calculations.

## Results

### Safranal Reduced Kidney Weight and the Somatic Index in MGN Rats

**Fig. 1:**
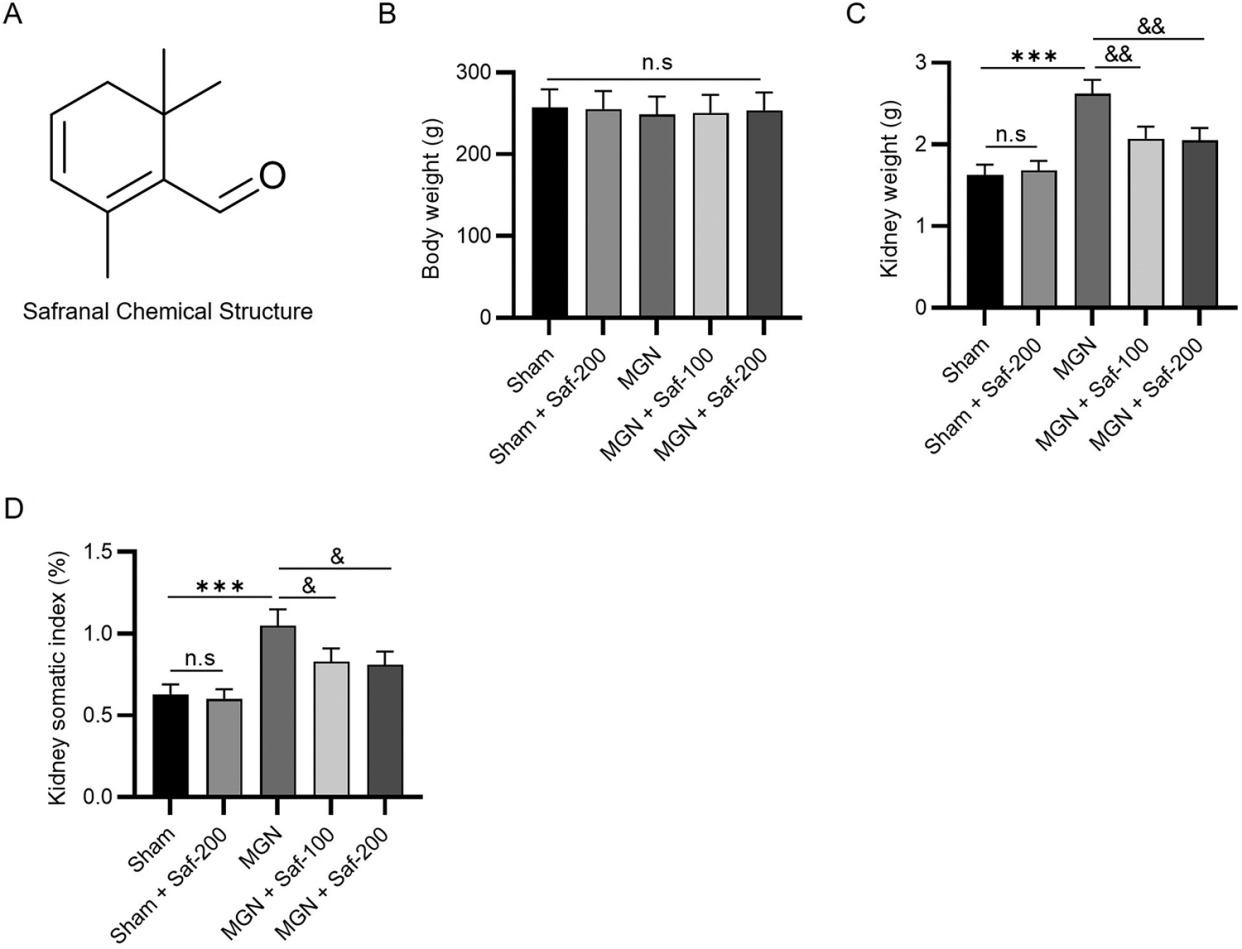
Safranal reduced kidney weight and the somatic index in MGN rats. **A** The chemical structure of safranal (C_10_H_14_O); **B** body weights of the rats in the sham, sham + Saf-200, MGN, MGN + Saf-100, and MGN + Saf-200 groups; **C** kidney weights of the rats; **D** kidney somatic index. ***P < 0.001 vs*.* the sham group, ^&^*P* < 0.05, ^&&^*P* < 0.01 *vs.* the MGN group. *n.s.* not significant. *n* = 3

A C-BSA-induced rat model of MGN was established to investigate whether safranal plays a renoprotective role. The chemical structure of safranal is shown in Fig. 1A. No significant changes in body weight were found among the 5 experimental groups, suggesting that neither the MGN model nor safranal treatment affected the body weight of the animals (Fig. 1B). In contrast, the kidney weight of MGN rats was significantly greater than that of sham rats (2.62 ± 0.17 g *vs*. 1.63 ± 0.12 g) (Fig. 1C). In addition, the elevation in kidney weight of model rats was notably reduced by 100 mg/kg safranal (2.07 ± 0.15 g) or 200 mg/kg safranal (2.05 ± 0.15 g) (Fig. 1C). There were no significant differences between the MGN + Saf-100 group and the MGN + Saf-200 group (Fig. 1C). Similarly, the kidney somatic index was greater in the MGN model group (1.05% ± 0.1%) than in the sham group (0.63% ± 0.06%). Safranal at 100 and 200 mg/kg significantly reduced the high index to 0.83% ± 0.08% and 0.81% ± 0.08%, respectively (Fig. 1D). No significant differences were detected between the MGN + Saf-100 group and the MGN + Saf-200 group (Fig. 1D), suggesting that the suppressive effect of safranal on the kidney somatic index did not occur in a dose-dependent manner. In addition, the sole administration of 200 mg/kg safranal to sham rats did not affect body weight, kidney weight or the kidney somatic index (Fig. 1B–D). Overall, the administration of safranal to MGN rats significantly lowered the kidney weight and somatic index.

### Safranal Improved Renal Dysfunction in MGN Rats

**Fig. 2:**
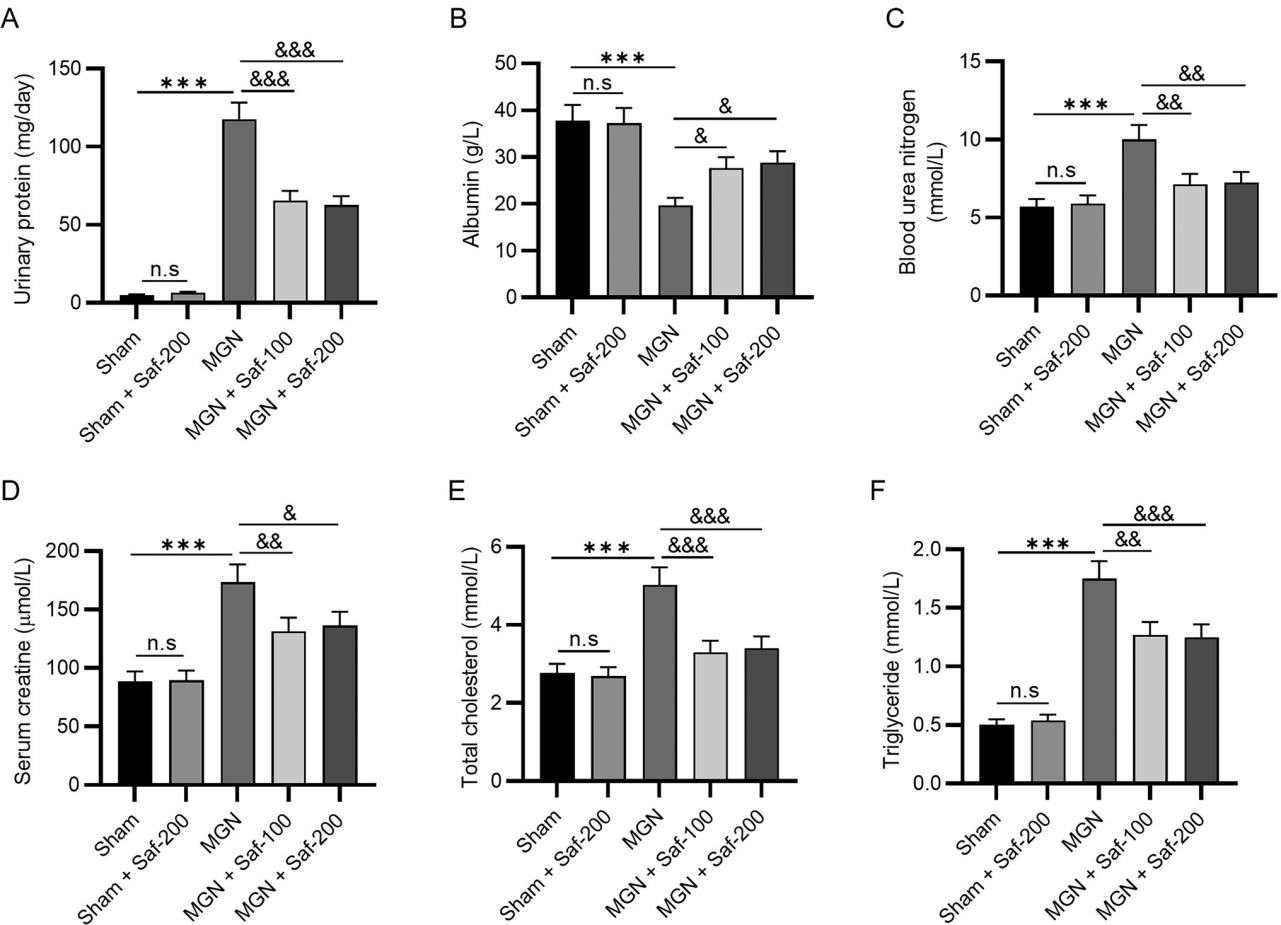
Safranal improved renal dysfunction in MGN rats. An automatic biochemical analyser was used to measure the urine protein level (**A**) and renal function parameters, including the levels of albumin (**B**), blood urea nitrogen (**C**), serum creatine (**D**), total cholesterol (**E**), and triglycerides (**F**), in the serum samples. ***P < 0.001 *vs.* the sham group; ^&^*P* < 0.05, ^&&^*P* < 0.01, ^&&&^*P* < 0.001 *vs.* the MGN group. *n.s.* not significant. *n* = 3

To evaluate renal dysfunction, the contents of renal function indicators in urine or serum samples were measured. The concentration of urinary protein increased to 117.68 ± 10.52 mg/day after modelling, and the alteration was suppressed in the presence of 100 mg/kg or 200 mg/kg safranal (65.63 ± 6.11 or 62.58 ± 5.71 mg/day) (Fig. 2A). In contrast, the concentration of albumin was markedly lower in the model rats than in the sham ones (19.64 ± 1.68 *vs.* 37.84 ± 3.32 g/L), and the change was reversed by 2 doses of safranal (27.63 ± 2.33 and 28.88 ± 2.37 g/L) (Fig. 2B). Compared with those in the sham rats, the levels of blood urea nitrogen, serum creatine, total cholesterol, and triglycerides in the serum samples of model rats were significantly greater (10.02 ± 0.93 *vs.* 5.68 ± 0.52 mmol/L, 173.28 ± 15.14 *vs.* 88.63 ± 8.32 μmol/L, 5.02 ± 0.46 vs. 2.77 ± 0.23 mmol/L, and 1.75 ± 0.15 *vs*. 0.5 ± 0.05 mmol/L, respectively), and these alterations were significantly repressed in the presence of safranal (Fig. 2C–F, *P* < 0.05, *P* < 0.01, *P* < 0.001). In addition, safranal alone did not significantly alter the levels of renal function indicators in sham rats (Fig. 2A–F). No significant differences were detected between the MGN + Saf-100 group and the MGN + Saf-200 group (Fig. 2A–F). Taken together, these findings suggest that safranal improves renal dysfunction in MGN rats by reducing urinary protein levels and improving biochemical parameters.

### Safranal Mitigated Renal Histological Changes and the Accumulation of IgG and C3

**Fig. 3:**
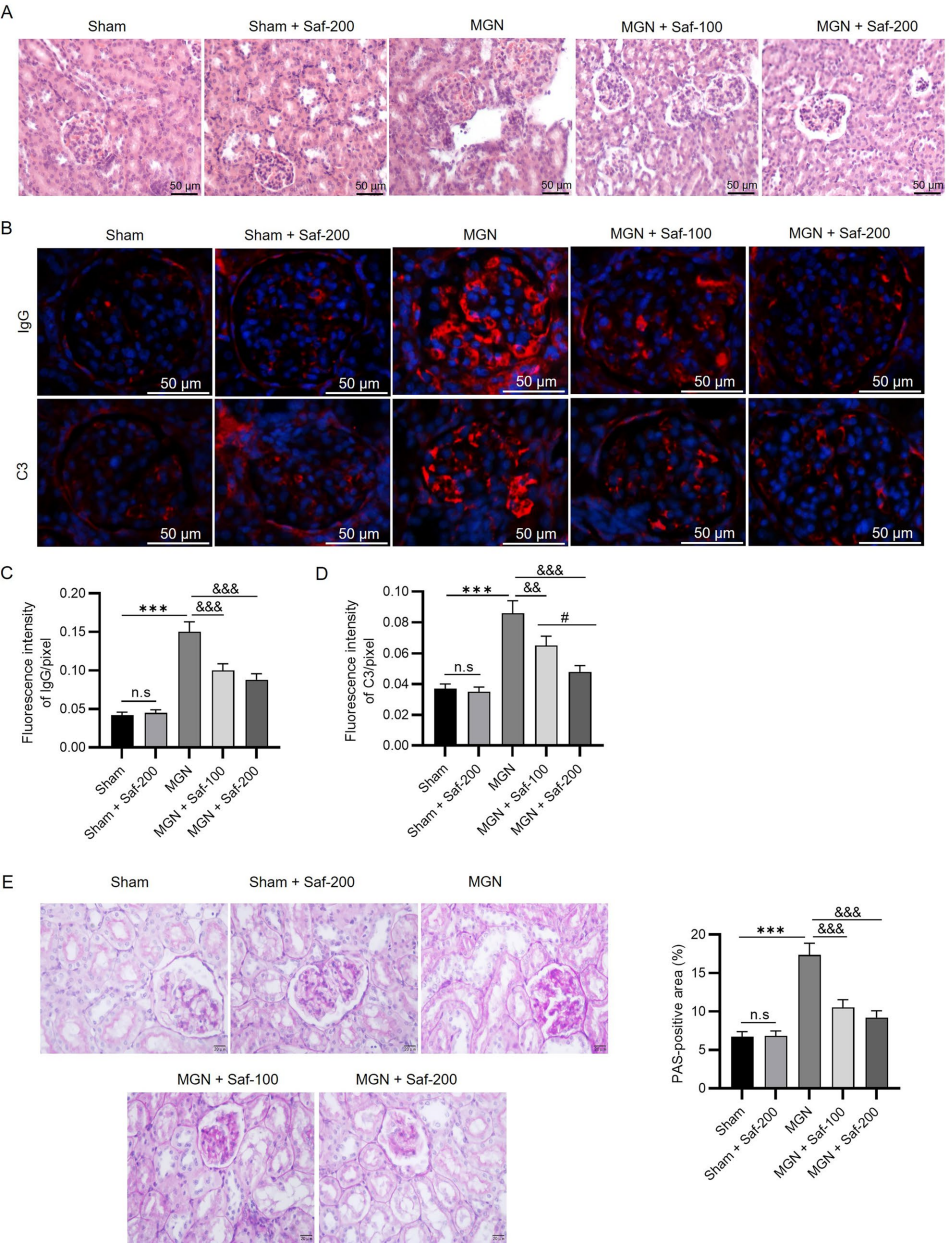
Safranal mitigated histological changes and the accumulation of IgG and C3. **A** H&E staining of kidney samples was performed to observe renal morphology (scale bar = 50 µm). **B** Immunofluorescence staining of kidney samples was performed to determine IgG and C3 expression (scale bar = 50 µm). **C** The fluorescence intensity of IgG was quantified. **D** Quantification of C3 fluorescence intensity. **E** Periodic acid-Schiff staining of kidney tissues was performed to assess pathological changes in the glomeruli (scale bar = 20 µm). ***P < 0.001 *vs.* the sham group; ^&&^*P* < 0.01, ^&&&^*P* < 0.001 *vs.* the MGN group. *n.s.* not significant. ^#^*P* < 0.05 *vs.* the MGN + Saf-100 group. *n* = 3

H&E staining was performed to observe renal morphology. Normal kidney tissue morphology was observed in the sham rats, with mesangia of normal size and intact basement membranes of the tubular epithelium and glomerular capillaries (Fig. 3A). Conversely, the glomerular basement membrane was thickened in MGN rats, accompanied by glomerular atrophy (Fig. 3A). The administration of safranal at 100 and 200 mg/kg inhibited abnormal structural changes, including glomerular expansion and inflammatory cell infiltration, in model rats (Fig. 3A). Immunofluorescence staining of kidney samples was then performed to measure C3 and Ig3 levels. As shown in Fig. 3B, C3 and IgG were particularly expressed along the capillary walls in the glomeruli of the model group. There was abundant deposition of C3 and IgG in the model group (C3: 0.086 ± 0.008, IgG: 0.15 ± 0.013) compared with the sham group (C3: 0.037 ± 0.003, IgG: 0.042 ± 0.004), and the trend was prominently reduced by the administration of safranal (Fig. 3B–D). In addition, there was no significant change in the IgG level between the MGN + Saf-100 group and the MGN + Saf-200 group (0.1 ± 0.009 and 0.088 ± 0.008) (Fig. 3C), whereas safranal reduced the C3 level (MGN + Saf-100: 0.065 ± 0.006, MGN + Saf-200: 0.048 ± 0.004) (Fig. 3D). The results of periodic acid-Schiff staining of renal tissues revealed that the thickness of the glomerular basement membrane was significantly greater in the MGN group than in the sham group (Fig. 3E). In addition, significant mesangial cell proliferation and mesangial matrix expansion were observed in the MGN group (Fig. 3E). Safranal at 100 or 200 mg/kg significantly diminished the thickness of the glomerular basement membrane and ameliorated mesangial hyperplasia in MGN model rats (Fig. 3E). The percentage of the PAS-positive area was significantly greater in the MGN group than in the sham group (17.36 ± 1.52% vs*.* 6.75 ± 0.63%), whereas the alterations were reversed by the administration of 100 and 200 mg/kg Saf (10.56 ± 0.99% and 9.23 ± 0.88%, respectively). There were no significant differences in the PAS-positive area between the MGN + Saf-100 group and the MGN + Saf-200 group (Fig. 3E). Overall, safranal improved renal morphology and reduced C3 and IgG accumulation in MGN rats, and safranal treatment alone did not significantly affect pathological changes in sham rats (Fig. 3A–E).

### Safranal Alleviated Podocyte Injury by Increasing Podocin and Nephrin Levels

**Fig. 4:**
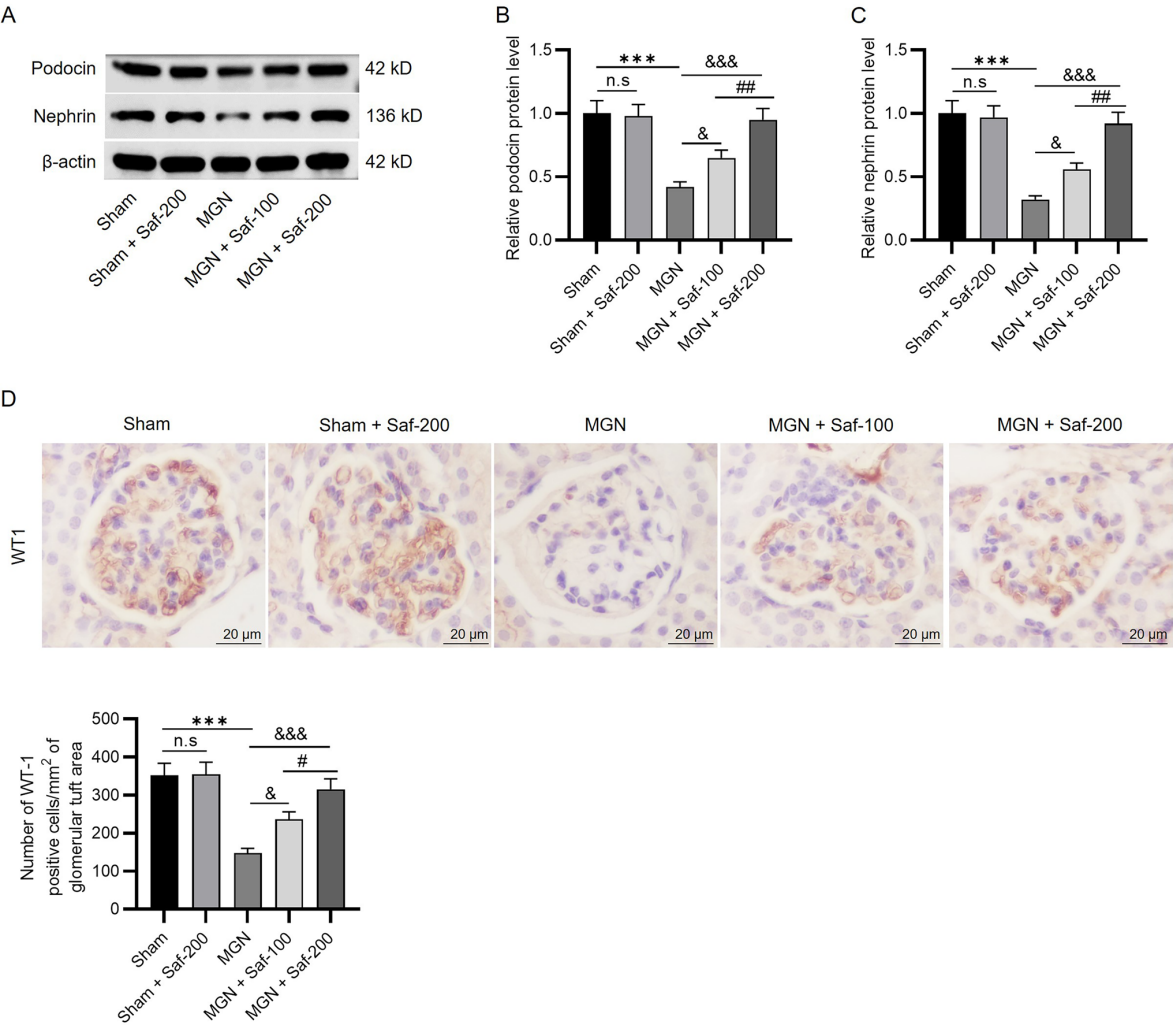
Safranal alleviated podocyte injury by increasing podocin and nephrin levels. **A** Western blot analysis was performed to measure the levels of podocyte proteins (podocin and nephrin) in renal tissue. **B**–**C** Quantification of podocin and nephrin levels. **D** Immunohistochemical staining of Wilms tumor protein-1 (WT-1) was performed to determine the number of podocytes in the glomerular tuft area (scale bar = 20 µm). ***P < 0.001 *vs.* the sham group, ^&^*P* < 0.05, ^&&&^*P* < 0.001 *vs.* the MGN group. *n.s.* not significant. ^#^*P* < 0.05, ^##^*P* < 0.01 *vs.* the MGN + Saf-100 group. *n* = 3

Podocin, a protein important for the maintenance of podocyte function, is a component of the slit diaphragm in the glomerular filtration barrier [6, 22]. Nephrin, a key component of the glomerular ultrafilter, can maintain a normal slit diaphragm in podocytes [23, 24]. Western blot analysis revealed that the protein levels of podocin and nephrin were significantly lower in the MGN group than in the sham group (podocin: 0.42 ± 0.04 *vs*. 1.0 ± 0.1; nephrin: 0.32 ± 0.03 *vs*. 1.0 ± 0.1), and the loss of these two podocyte proteins was ameliorated by safranal at 100 or 200 mg/kg (podocin: 0.65 ± 0.06 and 0.95 ± 0.09; nephrin: 0.56 ± 0.05 and 0.92 ± 0.09) (Fig. 4A‒C). WT-1 is a specific marker for podocytes and is highly expressed in podocytes. In adult kidneys, WT-1 can also be found in the nuclei of mature podocytes, indicating that WT-1 plays an essential role in sustaining podocyte function [25]. In this study, immunohistochemical staining of WT-1 was performed to determine the number of podocytes in the glomerular tuft area. The results revealed that the number of podocytes was markedly lower in the MGN group than in the sham group (148 ± 12 *vs.* 352 ± 32), and the decrease was obviously reversed by safranal administration (MGN + saf-100: 236 ± 20, MGN + saf-200: 315 ± 28) (Fig. 4D). In addition, there were no significant differences in podocyte marker levels or the number of podocytes between the sham group and the sham + Saf-200 group (Fig. 4A–D).

### Safranal Inhibited the Inflammatory Response by Regulating the Sirt1/NF-κB Pathway

**Fig. 5:**
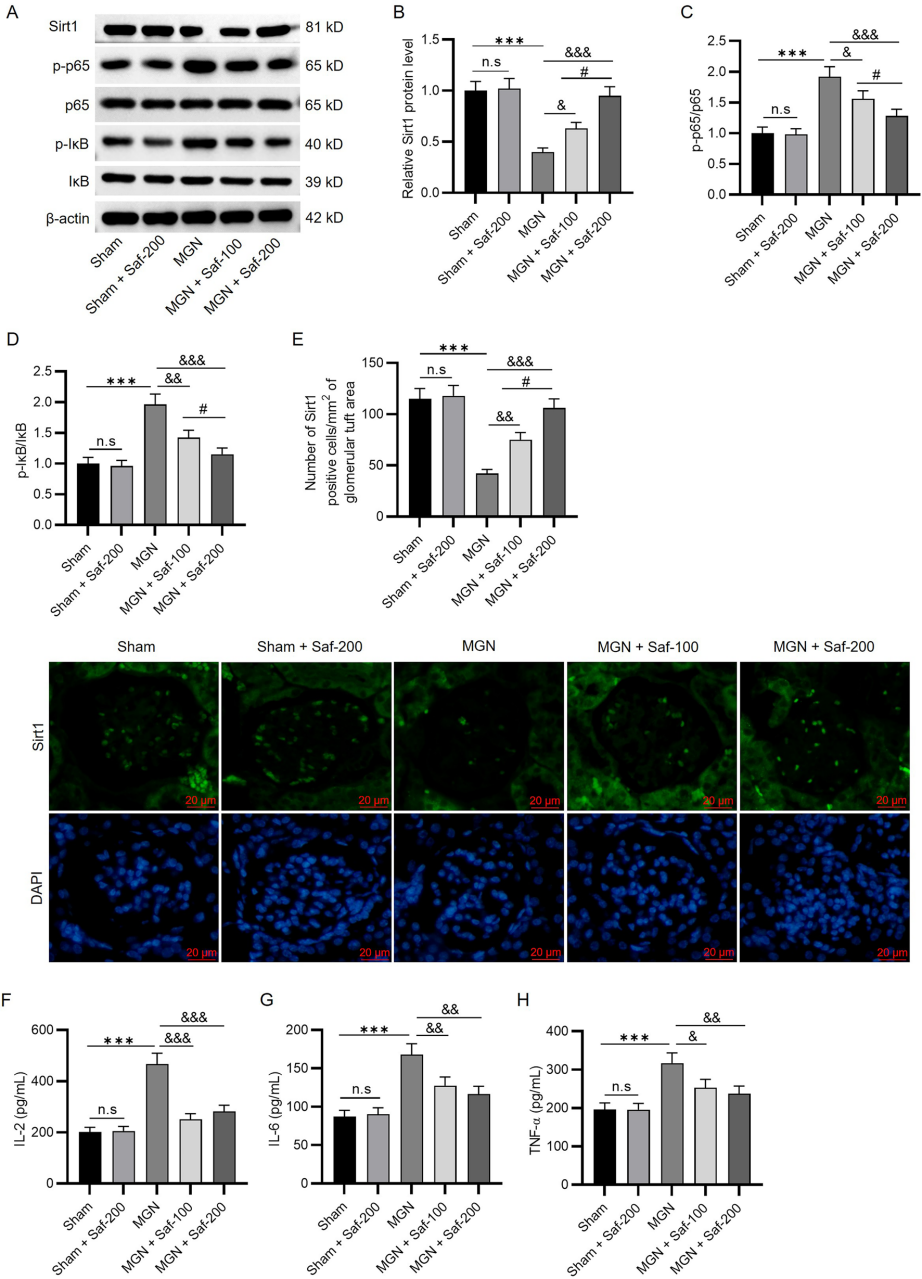
Safranal inhibited the inflammatory response by regulating the Sirt1/NF-κB pathway. **A** Western blotting was conducted to assess the protein levels of factors involved in the Sirt1/NF-κB pathway. Quantification of Sirt1 (**B**), p-p65/p65 (**C**), and p-IκB/IκB (**D**) levels; **E** immunofluorescence staining of Sirt1 was performed to measure Sirt1 expression in the kidney tissues of each group (scale bar = 20 µm). **F**–**H** ELISA was performed to measure the levels of inflammatory factors (IL-2, IL-6, and TNF-α) in renal homogenates. ***P < 0.001 *vs.* the sham group; ^&^*P* < 0.05, ^&&^*P* < 0.01, ^&&&^*P* < 0.001 *vs.* the MGN group. *n.s.* not significant. ^#^*P* < 0.05 *vs.* the MGN + Saf-100 group. *n* = 3

Sirt1/NF-κB signalling is closely related to kidney and podocyte injury in diabetic nephropathy [16, 26]. Nevertheless, the pathway involved in MGN has not yet been reported. As shown in Fig. 5A and B, the Sirt1 protein level was significantly lower in the kidney samples of MGN rats than in those of the sham group (0.4 ± 0.04 vs. 1 ± 0.09), and safranal effectively increased the Sirt1 protein level (MGN + Saf-100: 0.63 ± 0.06, MGN + Saf-200: 0.95 ± 0.09) relative to the Sirt1 expression level in the MGN group. The ratios of p-p65/p65 and p-IκB/IκB were noticeably increased in the MGN model group (1.92 ± 0.16 and 1.96 ± 0.17), and the activation of NF-κB signalling in MGN rats was suppressed in the context of safranal (Fig. 5A, C, D). These data verify that safranal enhances Sirt1 protein expression while reducing the levels of p65 and IκB phosphorylation. The results of immunofluorescence staining revealed that Sirt1 expression was significantly lower in the MGN group than in the sham group (42 ± 4 *vs*. 10 ± 3), and the alteration was improved by safranal treatment (MGN + Saf-100: 75 ± 7, MGN + Saf-200: 106 ± 9) (Fig. 5E). The findings further verified the promoting effect of safranal on Sirt1 signalling. ELISA was conducted to assess the concentrations of inflammatory factors in renal homogenates. IL-2, IL-6, and TNF-α levels were significantly greater in MGN rats (467.42 ± 42.15, 167.85 ± 14.23, and 317.42 ± 26.41 pg/mL) than in sham rats (202.53 ± 17.48, 87.11 ± 8.21, and 196.58 ± 16.52 pg/mL), and this trend was counteracted by safranal treatment (*P* < 0.05, *P* < 0.01, *P* < 0.001) (Fig. 5F–H). Taken together, these findings suggest that safranal inhibits inflammation in the kidneys of MGN rats by increasing Sirt1 expression and inhibiting NF-κB signalling.

### Safranal Activated Sirt1 Expression and Inhibited NF-κB Signalling to Regulate the Expression of Inflammatory Cytokines

**Fig. 6:**
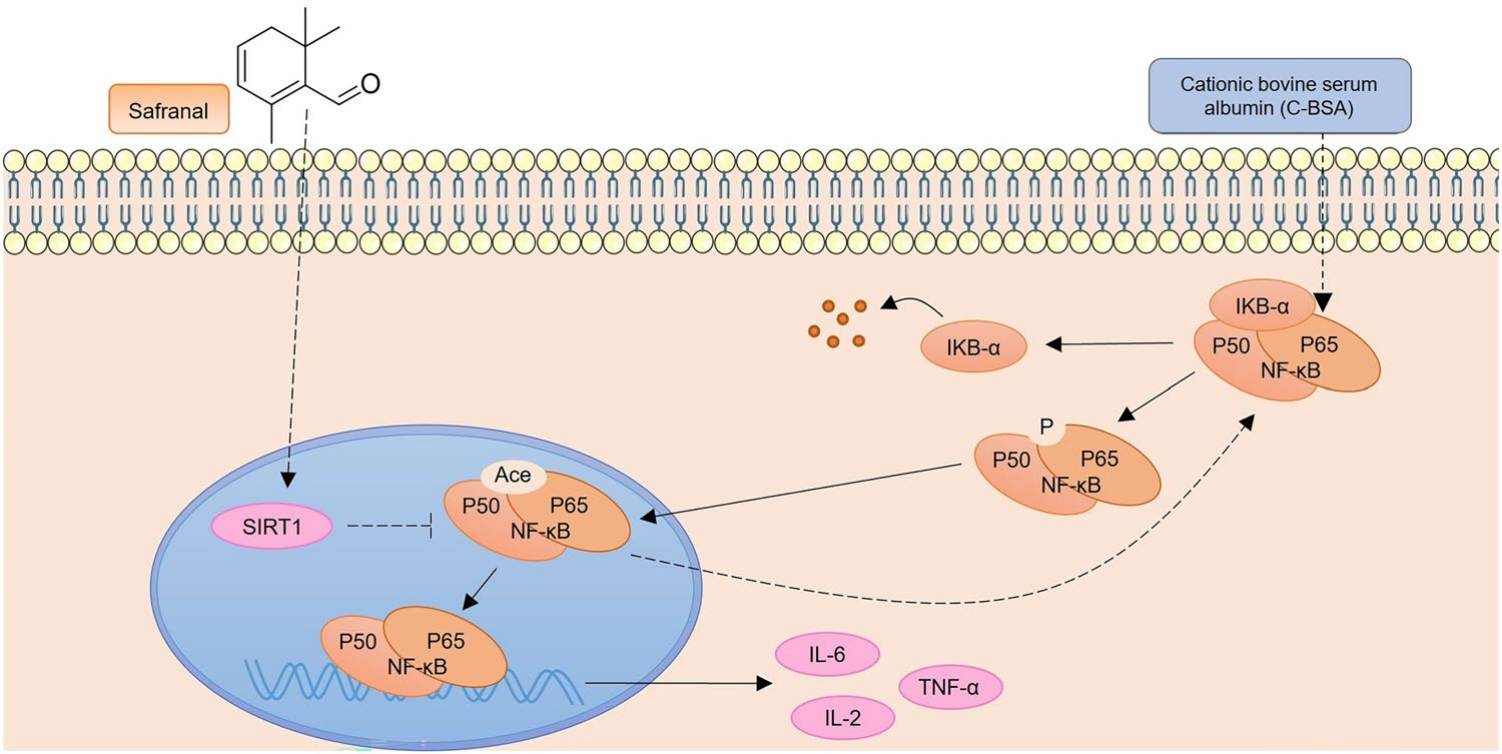
Safranal activated Sirt1 expression and inhibited NF-κB signalling to regulate the expression of inflammatory cytokines. A summary figure is provided to show the regulatory relationship between safranal and SIRT1/NF-κB signalling

A summary figure is provided to show the regulatory relationship between safranal and SIRT1/NF-κB signalling. As shown in Fig. 6, safranal activated Sirt1 and thereby inhibited NF-κB signalling by increasing p65 deacetylation and decreasing IκBα degradation in response to C-BSA stimulation. The summary was generated on the basis of our findings and those of previous studies [19, 27].

## Discussion

MGN developed exclusively in the animals that received intravenous administration of chemically modified C-BSA after being immunized with it [6]. In this study, an MGN rat model was successfully established, as suggested by high urine protein levels; dysregulated renal function parameters; inflammatory cell infiltration; thickened glomerular basement membrane; IgG and C3 deposition; and reduced levels of podocyte proteins (podocin and nephrin) in rat kidney samples. The results of this study demonstrate that safranal alleviates renal damage, inflammation, and podocyte injury in MRG rats by targeting the Sirt1/NF-κB pathway.

The presence of proteinuria is generally indicative of an impaired filtration membrane and reduced renal function [28]. In addition, hypoalbuminemia is a manifestation of nephrotic syndrome in MGN rats [2]. Blood urea nitrogen, serum creatine, total cholesterol, and triglycerides are important for maintaining the integrity of renal function and structure [29]. Histological analysis in this study verified the abnormal structure, such as glomerular expansion and inflammatory cell infiltration, in kidney samples. Therefore, the reduction in urine protein levels, recovery of albumin and renal function indicators, and improvement in glomerular damage after safranal administration indicate the protective role of safranal in MGN. This finding is consistent with the renoprotective role of safranal in other kidney diseases, such as diabetic nephropathy, ischaemia–reperfusion injury, and cisplatin-induced nephrotoxicity [12–14].

Podocytes are recognized as terminally differentiated cells within the glomerulus and feature regularly spaced foot processes that regulate glomerular filtration through the slit diaphragm [30]. Podocytes lack the capacity for cell division or replenishment, making podocyte injury crucial for renal prognosis [31]. Podocin and nephrin are key components of the slit diaphragm and maintain the integrity of the filtration barrier [32]. This study revealed that podocin and nephrin were downregulated after MGN modelling and that safranal protected against podocyte injury by upregulating these two factors, which is consistent with findings from previous articles focused on MGN [6, 33]. In contrast, Liu et al. [6] reported that, in addition to podocin and nephrin, desmin is also detected in renal tissues. It is an intermediate filament that is expressed in the glomerulus only in the event of podocyte damage, serving as a typical indicator of podocyte injury [6].

Safranal can directly induce the upregulation of Sirt1, as evidenced by previous studies [19, 34, 35]. Moreover, safranal interferes with NF-κB signalling by increasing Sirt1 expression to increase p65 deacetylation and inactivating IKK to decrease IκBα degradation [19]. NF-κB is the main transcription factor involved in inflammation-mediated nephropathy. According to previous studies, Cordyceps militaris fruiting bodies and diosgenin attenuate oxidative stress and inflammation via inactivation of the NF-κB pathway [2, 36]. Zhen-Wu-Tang attenuates kidney damage in MGN model rats by inhibiting NF-κB/NOD-like receptor protein 3 (NLRP3) inflammasome signalling or the AGE/RAGE/NF-κB pathway [6, 37]. This study is the first to report that safranal upregulated Sirt1 expression and inhibited the phosphorylation of p65 and IκB in the kidney tissue of MGN rats, which is consistent with the conclusions of a previous study focused on safranal [19]. Our data showed that safranal administration led to a significant reduction in the levels of inflammatory cytokines (IL-6, IL-2, and TNF-α). Despite its involvement in MGN, Sirt1/NF-κB signalling has also been reported to be involved in kidney function in other renal disorders. For example, the activation of Sirt1 and the inhibition of NF-κB signalling contribute to the improvement of renal function in 5/6 nephrectomy-induced chronic renal failure [38]. Quercetin alleviates renal inflammatory impairment in septic acute kidney injury by increasing Sirt1 expression through quenching NF-κB activation [39].

The available literature shows that safranal has a direct anti-inflammatory effect on various diseases. Like its role in kidney diseases, safranal also inhibits the macrophage-induced inflammatory response in colitis by reducing TNF-α and IL-6 levels via inactivation of the MAPK and NF-κB pathways [40]. In a rat model of nonalcoholic fatty liver disease, safranal significantly attenuated inflammation [41]. Safranal also ameliorates inflammation to protect neurons, thereby preventing memory and learning decline [42]. Moreover, as reported by Yang et al., safranal plays a neuroprotective role in Parkinson’s disease by repressing the activation of the NLRP3 inflammasome [43]. Gupta et al. further explained that safranal suppressed the activation of the NLRP3 inflammasome by preventing ASC oligomerization [44].

There are several limitations of this study. For example, further exploration of therapeutic targets such as microRNAs connecting safranal and the Sirt1/NF-κB pathway in MGN is lacking. In addition to the current signalling pathways, other potential signalling pathways that can be regulated by safranal may exist. Moreover, whether inhibiting Sirt1 signalling can affect the nephroprotective and anti-inflammatory activities of safranal has not been investigated.

In conclusion, safranal ameliorates kidney injury in MGN by mitigating the inflammatory response and improving podocyte injury via Sirt1/NF-κB signalling. This study indicates that safranal might be a promising and specific agent for improving renal function in patients with MGN. On the basis of available articles [5, 6, 44, 45], the regulatory effects of safranal on MAPK signalling and the NLRP3 inflammasome in MGN may be worthy of investigation in the future. In addition, sirt1 inhibitors such as nicotinamide and selisistat can be used [46, 47] to further verify the renoprotective role of safranal in MGN. Moreover, the influence of safranal on other biological processes, such as oxidative stress in MGN, could be a promising direction for future work, given its known antioxidant activity [48].

## Data Availability

The raw sequence data reported in this paper and detailed information will be available from the authors on request.
